# No evidence of hypercoagulability, altered fibrinolysis, or increased fibrin(ogen) microaggregates in never‐hospitalized long COVID

**DOI:** 10.1002/hem3.70501

**Published:** 2026-10-19

**Authors:** Emily Fraser, Kher Ng, Neda Hasan, Isobel Dunstan, Avianna Laws, Fraser Macrae, Jeries Abu‐Hanna, Robert Ariens, Victoria Harris, Jason Oke, Laura Saunders, James Grist, Guilhem Collier, Paul Hughes, Jim Wild, Roger Thompson, Fergus Gleeson, Susan Shapiro, the EXPLAIN Investigators

**Affiliations:** ^1^ Respiratory Medicine Department Oxford University Hospitals NHS Foundation Trust Oxford United Kingdom; ^2^ Radcliffe Department of Medicine Oxford University Oxford United Kingdom; ^3^ Pharmacology Department Oxford University Oxford United Kingdom; ^4^ Oxford Radiology Research Unit Oxford University Hospitals NHS Foundation Trust Oxford United Kingdom; ^5^ Discovery and Translational Science Department Leeds Institute of Cardiovascular and Metabolic Medicine, School of Medicine, University of Leeds Leeds United Kingdom; ^6^ Nuffield Department of Primary Care Health Sciences Oxford University Oxford United Kingdom; ^7^ POLARIS, Division of Clinical Medicine School of Medicine and Population Health, University of Sheffield Sheffield United Kingdom; ^8^ Oxford Haemophilia and Thrombosis Centre, Oxford University Hospitals NHS Foundation Trust Oxford United Kingdom

Long COVID (LC), or post‐COVID‐19 condition (PCC), is a heterogenous disorder characterized by persistence of symptoms >12 weeks after COVID‐19 infection that cannot be explained by alternative causes.^1^ Common symptoms include breathlessness, fatigue and cognitive difficulties.^1^, ^2^ Although hospitalization with acute COVID‐19 increases the risk of LC, the majority of people with LC were never hospitalized.^2^

An international research effort to understand the biological mechanisms driving symptom persistence, including breathlessness, has not identified a single or unifying explanation although most pathways converge on a dysregulated inflammatory response.^3^ A prominent hypothesis is that individuals with LC are prothrombotic and develop “microclots” that lodge in the peripheral vasculature causing local tissue hypoxia and symptoms including breathlessness and post‐exertional malaise.^3^ This hypothesis stems from observations in acute COVID‐19 of a hypercoagulable state with impaired fibrinolysis and increased thrombotic risk, together with reports of amyloid‐fibrin(ogen) microaggregates in LC.^4^, ^5^, ^6^ This prothrombotic hypothesis has been used to justify the use of antithrombotic medications and plasmapheresis outside of clinical trials, despite these treatments having established adverse effects.^6^, ^7^ However, the hypothesis had not been robustly tested in well‐defined never‐hospitalized cohorts with specialist‐confirmed LC. We therefore investigated whether established markers of coagulation/fibrin formation, platelet and endothelial activation, NETosis (neutrophil extracellular traps) and fibrinolysis, together with fibrin clot architecture and fibrin(ogen) microaggregates, were associated with symptoms, pulmonary gas transfer or perfusion abnormalities in participants with never‐hospitalized LC.

Participants were recruited through four UK specialist LC centers to a substudy of the EXPLAIN observational clinical trial (hypErpolarized Xenon magnetic resonance puLmonAry ImagiNg in patients with LC; ISRCTN14304264).^8^ Eligible individuals were adults who had never been hospitalized with acute COVID‐19. LC was diagnosed according to NICE criteria after multidisciplinary assessment and exclusion of alternative causes.^1^ Participants were classified clinically as breathless LC (BLC) or non‐breathless LC (NBLC). We excluded current smokers, those with significant chest CT abnormalities, airflow obstruction (FEV1/FVC < 0.7), and those receiving anticoagulants or antiplatelet therapy. Age‐ and sex‐matched controls had prior confirmed COVID‐19 but were fully recovered.

For the hemostasis substudy, blood was collected in 2.7 mL blue citrate (3.2%) BD Vacutainer™ tubes using standard venepuncture technique. Platelet‐poor plasma was obtained by centrifugation of whole blood samples for 15 min at 3000 × *g* at room temperature for 15 min, aliquoted, and frozen at −80°C within 4 h of collection. Blood and urine were analyzed centrally for markers of coagulation/fibrin formation (D‐dimer, Clauss fibrinogen), endothelial activation (von Willebrand factor [VWF], soluble thrombomodulin), platelet activation (P‐selectin, soluble CD40 ligand (sCD40L), urinary 11‐dehydro‐thromboxane B2), NETosis (myeloperoxidase and citrullinated histone H3) and fibrinolysis (PAI‐1; 50% clot lysis time). A subset underwent blinded confocal analysis of fibrin fiber density and fibrin(ogen) microaggregates using a previously reported quantifiable technique.^9^ Dissolved‐phase hyperpolarized xenon Magnetic Resonance Imaging (HPX‐MRI) and dynamic contrast‐enhanced lung MRI (DCE‐MRI) assessed gas transfer and pulmonary perfusion, respectively. Further methods are provided in the Supporting Information S4: Supplementary Digital Content (SDC).

A total of 177 participants consented to this EXPLAIN substudy; 26 were excluded, leaving 151 for plasma and/or urine biomarker analysis (Supporting Information S1: Figure 1). Fibrin fiber and fibrin(ogen) microaggregate analyses were undertaken in 55 samples, randomly selected from within the BLC, NBLC and control groups (Supporting Information S1: Figure 1).

Age, sex, body mass index (BMI) and smoking status were not significantly different between groups (Table 1). Time from infection to sampling was longer in BLC than NBLC and controls (median 596 days vs. 483 and 558 days, respectively, P < 0.01) (Table 1). Symptom burden was substantial; dyspnea‐12 was higher in BLC than NBLC group (P < 0.01), but there were no significant differences between these groups for fatigue (FACIT‐F), anxiety (GAD‐7), and general health status (EuroQoL‐VAS) (Supporting Information S3: Table 1). Conventional pulmonary function tests showed only small differences, and although mean FEV_1_ *z*‐score and the gas transfer values TL_CO_ and K_CO_ were slightly lower in BLC than NBLC and controls, there were no significant differences in the proportion of individuals with abnormal *z*‐scores (i.e., below −1.64) (Supporting Information S3: Table 1).

**Table 1:** Demographics, clinical characteristics, and prothrombotic biomarkers in long COVID (LC) participants (breathless and non‐breathless) and controls.

	Breathless (*n* = 80)	Non‐breathless (*n* = 37)	Control (*n* = 34)	P‐value
*Demographics and clinical characteristics*				
Female	50 (62.5%)	24 (64.9%)	25 (73.5%)	0.52
Age (years)	48.2 ± 12.5	46.2 ± 9.3	47.4 ± 13.1	0.51
BMI (kg/m^2^)	29.1 ± 6.1	28.1 ± 5.3	29.6 ± 6.2	0.49
Ethnicity				
White	71 (89%)	37 (100%)	27 (79%)	**0.01**
Non‐White	9 (11%)	0 (0%)	7 (21%)	
Days post‐COVID infection	596 (468–967)	483 (329–655)	558 (488–662)	**0.01**
Vaccinated	74 (92.8%)	37 (100.0%)	34 (100.0%)	0.08
Asthma	8 (9.6%)	3 (8.1%)	7 (20.6%)	0.21
Diabetes	3 (3.8%)	2 (5.4%)	1 (2.9%)	0.87
Smoking				
Never	56 (71.1%)	28 (75.7%)	27 (79.4%)	0.55
Former	24 (28.9%)	9 (24.3%)	7 (20.6%)	
*Prothrombotic biomarkers*				
Coagulation/fibrin formation				
Fibrinogen (g/L)	2.90 (2.50–3.30)	3.00 (2.60–3.30)	2.80 (2.60–3.38)	0.75
D‐dimer (µg/L FEU)	240 (190–393)	196 (190–306)	224 (190–309)	0.26
Endothelial activation				
Von Willebrand factor (IU/mL)	0.92 (0.70–1.18)	0.85 (0.67–1.20)	0.93 (0.73–1.18)	0.64
Soluble thrombomodulin (ng/mL)	4.13 (3.60–4.74)	4.15 (3.49–4.51)	4.08 (3.76–4.36)	0.74
Platelet activation				
sCD40L (ng/mL)	0.10 (0.08–0.13)	0.11 (0.09–0.14)	0.13 (0.09–0.15)	**0.02**
P‐selectin (ng/mL)	38.28 (32.72–46.24)	37.58 (31.39–45.18)	40.69 (34.82–47.45)	0.75
Urinary 11‐dhTXB_2_/creatinine (pg/mg)	2755.7 (2040.1–3623.6)	2840.8 (1503.5–3618.4)	3101 (2249–4027)	0.32
NETosis				
Myeloperoxidase (ng/mL)	13.49 (10.57–17.81)	13.28 (11.44–15.80)	15.27 (13.51–18.66)	0.17
Citrullinated H3 (ng/mL)	0.32 (0.18–0.57)	0.23 (0.15–0.45)	0.30 (0.16–0.51)	0.63
Fibrinolysis				
PAI‐1 (ng/mL)	8.92 (6.56–11.79)	7.39 (4.64–10.28)	8.34 (5.25–11.85)	0.24
50% clot lysis time (min)	43.81 (38.73–51.67)	41.68 (37.12–47.32)	40.53 (35.58–48.35)	0.27

*Note*: Parametric data expressed in mean ± standard deviation; non‐parametric data expressed in median (interquartile range). P‐values derived from analysis of variance (ANOVA) for parametric data, Kruskal–Wallis for non‐parametric data. Bold values indicate statistically significant at P ≤ 0.05.

Abbreviations: 11‐dhTxB2, 11‐dehydro‐thromboxane B2; BMI, body mass index; NET, neutrophil extracellular trap; PAI‐1, plasminogen activator inhibitor‐1.

Pulmonary perfusion and gas transfer measures did not show significant differences between groups. HPX‐MRI measures, RBC:Gas and RBC:M (assessing abnormalities at the alveolar‐capillary membrane and pulmonary vasculature), RBC:M CoV (reflecting ^129^Xe diffusion heterogeneity), and DCE‐MRI mean transit time (a measure of pulmonary perfusion) were comparable (Supporting Information S3: Table 1). Routine laboratory blood tests were within normal ranges in all participants (Supporting Information S3: Table 2).

Prothrombotic and fibrinolytic markers showed no differences between groups: D‐dimer, Clauss fibrinogen, VWF, soluble thrombomodulin, P‐selectin, urinary 11‐dehydro‐thromboxane B2, myeloperoxidase or citrullinated histone H3, PAI‐1, and 50% clot lysis (Table 1). Although sCD40L was slightly lower in NBLC and BLC than controls, statistical significance was lost when the BLC and NBLC groups were combined (Supporting Information S3: Table 3). Furthermore, as acute COVID‐19 and platelet activation are associated with increased sCD40L,^10^ this finding of possible reduced sCD40L in NBLC does not support persistent prothrombotic activation in NBLC.

Correlation analysis showed no strong associations between these prothrombotic markers and health‐related questionnaires or pulmonary function assessments (Supporting Information S2: Figure 2). Weak correlations were observed but were inconsistent across related physiological measures. For example, PAI‐1 correlated positively with dyspnea‐12, and negatively with FACIT‐F (where lower scores indicate worse fatigue) and RBC:M, but not with other pulmonary function parameters.

For the fibrin fiber and microaggregate analyses, demographics were similar to the overall groups (Supporting Information S3: Table 4). Mean fibrin fiber density was 47.9 ± 15.0 fibers/100 µm in controls, 43.6 ± 12.3 in BLC, and 38.0 ± 11.0 in NBLC, with no significant difference between groups (P = 0.22) (Supporting Information S3: Table 5). There remained no significant difference when BLC and NBLC were combined (Supporting Information S3: Table 6). The majority of plasma samples had multiple fibrin(ogen) microaggregates, but neither their number nor area differed between BLC, NBLC and controls (Figure 1, Supporting Information S3: Table 4). The number of larger microaggregates (>100 µm^2^) was also comparable (Figure 1, Supporting Information S3: Table 5), with unchanged findings when all LC participants were analyzed collectively versus controls (Supporting Information S3: Table 6).

**Figure 1:**
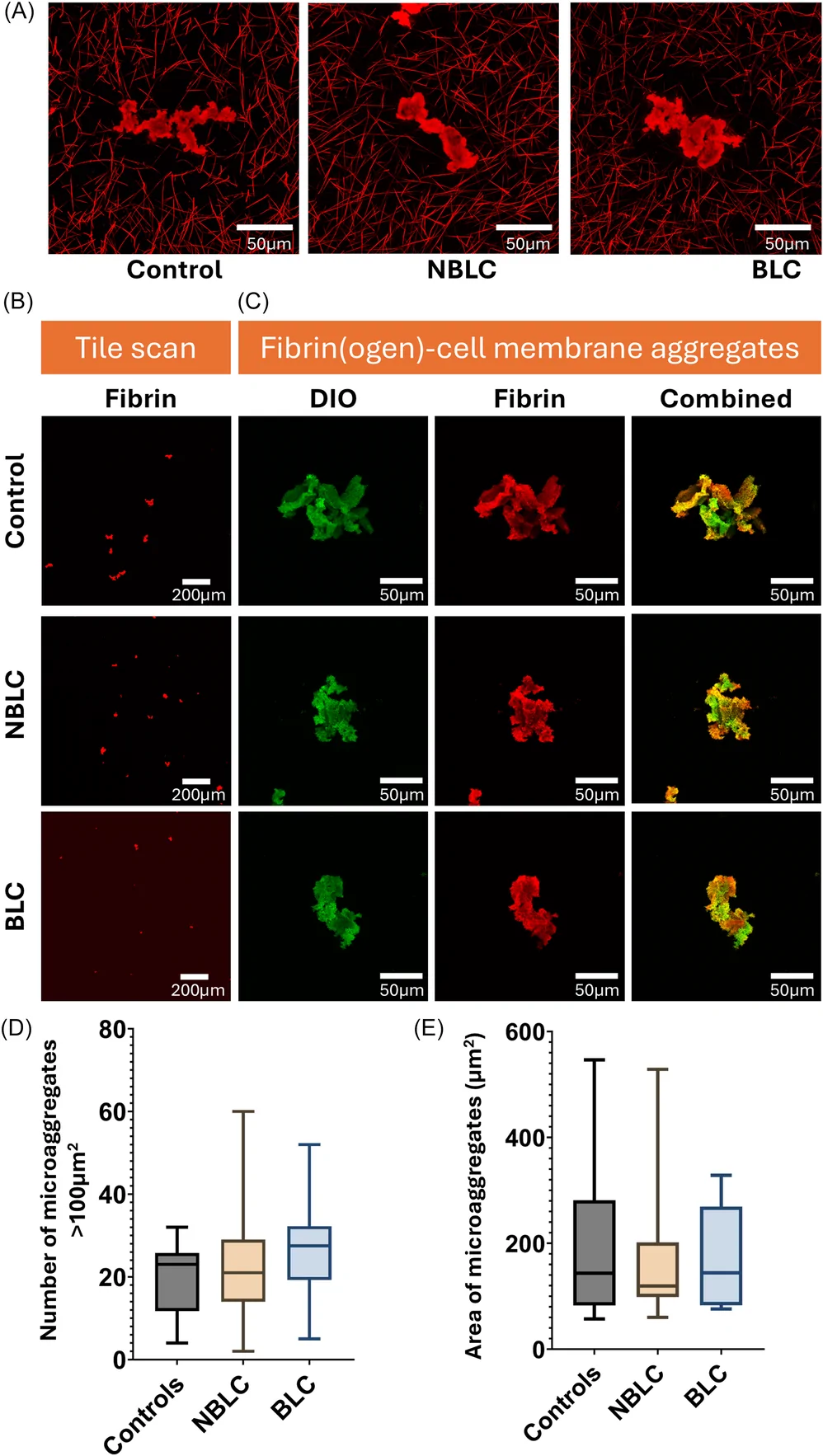
**(A) Fibrin network**. Representative *z*‐stack confocal images to illustrate the fibrin network across participant groups. **(B–E)** Fibrin(ogen) microaggregate analysis. **(B)** Representative confocal “tile” images to illustrate the presence of fibrin(ogen) microaggregates across all three groups. **(C)** Representative confocal images of fibrin(ogen) microaggregates across all three groups. **(D)** Box plots showing the quantified comparison of the total number of fibrin(ogen) microaggregates greater than 100 µm^2^ in size. **(E)** The average area of the fibrin(ogen) microaggregates; no significant difference in P‐values (analysis of variance [ANOVA] test). Key: fibrin‐red; cell membranes—green. BLC, breathless long COVID; NBLC, non‐breathless long COVID.

To our knowledge, this is the first study to undertake a comprehensive panel of prothrombotic and fibrinolytic biomarkers with quantitative fibrin structure analysis in a well‐characterized group of never‐hospitalized individuals with LC. Our findings do not support the presence of a persistent systemic prothrombotic state as an explanation for chronic breathlessness or other LC symptoms in this population, sampled a median of 483 days after infection in NBLC and 596 days in BLC.

Previous studies supporting persistent prothrombotic abnormalities have predominantly examined follow‐up cohorts after hospitalization with acute COVID‐19 rather than specialist‐confirmed LC. Persistently increased VWF, factor VIII, and soluble thrombomodulin have been reported at median 68 days after acute infection, without correlation to symptoms/6‐min walk test results (*n* = 50).^11^ Slightly increased PAI‐1 and prolonged clot lysis were reported 12 months after hospitalization with acute COVID‐19 (*n* = 44); however, participants and controls were not matched for BMI (which influences PAI‐1), and these hemostatic markers did not correlate with symptoms.^12^, ^13^ Raised NETosis markers were reported >6 months after acute COVID‐19 requiring intensive care (*n* = 42),^14^ and endothelial/inflammatory signatures have been described in LC (*n* = 25) although the control group demographics were not specified.^15^ Raised VWF antigen:ADAMTS13 activity (suggestive of ongoing endotheliopathy), measured a median of 6 months following acute infection, correlated with impaired exercise capacity.^16^ In contrast, our participants had specialist‐confirmed LC diagnosis, had never been hospitalized, were studied substantially later after acute infection, and were compared to well‐matched controls.

Fibrin structure in LC has not previously been reported. Fibrin clot structure is directly impacted by fibrinogen and thrombin concentrations, and dense fibrin networks of thin fibers have been associated with both arterial and venous thromboembolic disease.^17^ In contrast to severe COVID‐19,^18^ there was no significant difference in fibrin fiber density between groups.

We also investigated for the recently described phenomenon of fibrin(ogen) microaggregates. The presence of amyloid‐fibrin(ogen) microaggregates in LC was initially reported by the Pretorius group using thioflavin T fluorescent microscopy.^4^, ^5^ Subsequently, using a quantitative flow cytometry technique, the group reported increased size and number of amyloid‐fibrin(ogen) microaggregates post‐COVID‐19.^19^ Similar aggregates have been reported in non‐COVID intensive care patients where proteomic analysis demonstrated predominantly fibrinogen, inflammatory proteins and lipoproteins.^20^ Meanwhile, the Ariens group reported fibrin(ogen)‐cell membrane microaggregates after acute pulmonary embolus.^9^ We chose to study fibrin(ogen)‐cell membrane microaggregates as these structures are representative of known clot constituents. We used the previously reported technique^9^ and quantitative blinded analysis. We counted aggregates >5 µm^2^, as smaller aggregates were unlikely to directly occlude capillaries, and additionally analyzed large aggregates >100 µm^2^. Neither measure differed between LC and controls. Differences from previous reports may reflect cohort definition (specialist‐confirmed LC as opposed to self‐declared), severity of acute COVID‐19 or timing of sampling. Increased fibrin(ogen) microaggregates after COVID‐19 could potentially represent residual inflammatory responses rather than a phenomenon specific to LC especially as fibrin(ogen) microaggregates have been found in other conditions and healthy volunteers.^20^ The description of fibrin(ogen) microaggregates as “microclots” has been questioned.^6^, ^7^ Furthermore, their clinical significance remains unclear, especially as they have been reported in other conditions as well as healthy volunteers, and they could be an artifact of plasma processing.^7^, ^9^, ^20^

Two interventional randomized controlled trials have recently been published which evaluated treatments based on the “prothrombotic” hypothesis of LC.^6^, ^7^ STIMULATE‐ICP was an open‐label adaptive platform trial of adults with never‐hospitalized LC; it found no benefit of the anticoagulant rivaroxaban, 10 mg taken daily for 12 weeks, on self‐reported fatigue.^21^ Plasma exchange has been proposed as a potential treatment as hypothetically it could remove “microclots/microaggregates” from the circulation; a double‐blind placebo‐controlled trial found no benefit in LC.^22^

Strengths of this study include: participants had specialist‐confirmed LC with substantial symptom burden, demographics similar to a large UK LC population study,^23^ a long time from infection to assessment with comprehensive pulmonary testing, a broad panel of validated hemostasis assays, and quantitative fibrin(ogen) microaggregate analysis. As never‐hospitalized individuals comprise about 90% of people with LC,^1^, ^2^ these findings should be relevant to the majority of the LC population.

Limitations include single time‐point sampling (although all LC participants had significant persistent symptom burden) and differences in median time from acute infection between BLC, NBLC and controls (although as no measured biomarker differed between groups, we do not think that this impacted the results). The population studied was largely Caucasian. Missing data occurred in questionnaires, lung function tests, HPX‐MRI, and perfusion MRI due to participants' incomplete participation and issues related with image acquisition, quality, and processing. Classification into BLC and NBLC is imperfect given the complex and fluctuating nature of LC breathlessness; associations between symptoms, lung function parameters and biomarkers were therefore assessed with the LC group collectively. All the hemostatic tests were undertaken on samples that had been frozen (freeze–thaw cycles were minimized and uniform across assays). Fibrin and fibrin(ogen) microaggregate analyses were only performed in a subgroup due to time limitations. The precise composition of the fibrin(ogen)‐cell membrane aggregates is not known. Further work is required to establish whether different techniques are detecting equivalent fibrin(ogen) microaggregates (amyloid vs. cell membrane), and whether they are an artifact of blood processing or are of clinical significance. Lastly, the findings of this study are not necessarily generalizable to those with LC following hospitalization with acute COVID‐19.

In summary, in never‐hospitalized patients with specialist‐confirmed LC, we found no evidence of persistent prothrombotic activation, impaired fibrinolysis, abnormal fibrin fiber architecture or increased fibrin(ogen) microaggregates, and no meaningful associations with breathlessness, fatigue or pulmonary perfusion and gas transfer metrics. These findings suggest that antithrombotic therapy is not justified outside of clinical trials for never‐hospitalized LC or in the absence of a separate recognized indication such as venous or arterial thrombosis.

## AUTHOR CONTRIBUTIONS

**Emily Fraser**: Conceptualization; methodology; supervision; data curation; formal analysis; funding acquisition; writing—original draft; writing—review and editing. **Kher Ng**: Methodology; investigation; formal analysis; writing—review and editing. **Neda Hasan**: Methodology; investigation; formal analysis; writing—review and editing. **Isobel Dunstan**: Formal analysis; data curation; project administration; investigation. **Avianna Laws**: Project administration; investigation. **Fraser Macrae**: Methodology; investigation; formal analysis; supervision; writing—review and editing. **Jeries Abu‐Hanna**: Methodology; supervision; investigation; writing—review and editing. **Robert Ariens**: Conceptualization; methodology; supervision; writing—review and editing. **Victoria Harris**: Investigation; methodology; formal analysis; writing—review and editing. **Jason Oke**: Data curation; formal analysis; writing—review and editing. **Laura Saunders**: Methodology; investigation; writing—review and editing. **James Grist**: Methodology; investigation; writing—review and editing. **Guilhem Collier**: Methodology; investigation; writing—review and editing. **Paul Hughes**: Methodology; investigation; writing—review and editing. **Jim Wild**: Methodology; investigation; writing—review and editing. **Roger Thompson**: Methodology; investigation; supervision; formal analysis; writing—review and editing. **Fergus Gleeson**: Conceptualization; methodology; investigation; writing—review and editing. **Susan Shapiro**: Conceptualization; methodology; investigation; formal analysis; supervision; writing—original draft; writing—review and editing.

## CONFLICT OF INTEREST STATEMENT

The authors declare no conflicts of interest.

## ETHICS STATEMENT

This study was approved by the South Central Research Ethics Committee (21/SC/0398) and International Standard Randomized Controlled Trial Number (ISRCTN14304264).

## FUNDING

The study was funded by the National Institute for Health and Care Research (NIHR) (COV‐LT2‐0049), with additional funding from NIHR Sheffield Biomedical Research Centre (NIHR203321), the NIHR Oxford Biomedical Research Centre, Medical Research Council (grant MR/M008894/1), and INSIGNEO institute for in silico medicine. S.S. received funding support from the Medical Research Council (MR/T024054/1). F.M. is supported by the Wellcome Trust (215861/Z/19/Z) and British Heart Foundation (PG/25/12256). The RASA Laboratory is supported by British Heart Foundation Program and Project grants (RG/F/23/110112 and PG/23/11184). RASA is supported in part by the National Institute for Health and Care Research (NIHR) Leeds Biomedical Research Centre.

## Supporting information


**Figure S1.** Participant recruitment and numbers in biomarker, lung function, fibrin network, and fibrin(ogen) microaggregate analyses.

**Figure S2.** Plots to examine potential associations between prothrombotic biomarkers with symptoms measured via patient‐reported outcome measures (dyspnoea‐12, FACIT‐F, and EuroQoL‐VAS), gas transfer (TL_CO_ *z*‐score), and HPX‐MRI metrics of diffusion (RBC:M mean *z*‐score, RBC:Gas mean *z*‐score), and perfusion MRI measures of pulmonary mean transit time (MTT in seconds). Abbreviations: 11‐dhTxB_2_, 11‐dehydro‐thromboxane B_2_; FACIT‐F, functional assessment of chronic illness therapy‐fatigue; MPO, myeloperoxidase; PAI‐1, plasminogen activator inhibitor‐1; RBC:Gas, red blood cell to gas ratio; RBC:M, red blood cell to membrane ratio; sCD40L, soluble CD40 ligand; TL_CO_, diffusing capacity of the lungs for carbon monoxide; VAS, visual analog scale.

**Supporting Information**.

**Supporting Information**.

## Data Availability

The data that support the findings of this study are available from the corresponding author upon reasonable request.
