# Supplementary material for: No evidence of hypercoagulability, altered fibrinolysis, or increased fibrin(ogen) microaggregates in never‐hospitalized long COVID
Source: Hemasphere. 2026 Oct 19;10(10):e70501. doi: 10.1002/hem3.70501 (PMC13680132; doi:10.1002/hem3.70501)
Supplement: Supplementary file 1 [file HEM3-10-e70501-s001.docx]

**Figure S1.** Participant recruitment and numbers in biomarker, lung function, fibrin network, and fibrin(ogen) microaggregate analyses.
