# Supplementary material for: No evidence of hypercoagulability, altered fibrinolysis, or increased fibrin(ogen) microaggregates in never‐hospitalized long COVID
Source: Hemasphere. 2026 Oct 19;10(10):e70501. doi: 10.1002/hem3.70501 (PMC13680132; doi:10.1002/hem3.70501)
Supplement: Supplementary file 2 [file HEM3-10-e70501-s002.docx]

| QUESTIONNAIRES | Breathless LC  (n=75) | Non-breathless LC (n=35) | | - | *P* value |
| --- | --- | --- | --- | --- | --- |
| Fatigue FACIT-F | 24.0 (15.0 – 29.50) | 22.0 (16.0 – 30.5) | | - | 0.87 |
| Dyspnoea-12 | 12.0 (8.0 – 18.0) | 2.0 (0.0 – 6.0) | | - | **<0.01** |
| EQ-5D-5L Health VAS | 60.0 (50.0 – 70.0) | 60.0 (50.0 – 70.0) | | - | 0.76 |
| GAD-7 | 6.0 (2.0 – 10.0) | 4.0 (2.5 – 6.5) | | - | 0.20 |
| **LUNG FUNCTION DATA** | **Breathless LC (n=79)** | **Non-breathless LC**  **(n=36)** | | **Control**  **(n=34)** |  |
| TL_CO_ z-score | -0.83 ± 0.92 | | -0.26 ± 0.97 | -0.13 ± 0.96 | **<0.01** |
| Abnormal TL_CO_ z-score* | 14 (17.7%) | | 3 (8.3%) | 2 (5.9%) | 0.19 |
| K_CO_ z-score | -0.08 ± 1.00 | | 0.22 ± 0.92 | 0.56 ± 1.09 | **<0.01** |
| Va z-score | -0.94 ± 1.01 | | -0.60 ± 0.93 | -0.79 ± 1.11 | 0.25 |
| FEV_1_ z-score | -0.10 ± 0.89 | | 0.38 ± 1.03 | 0.11 ± 0.87 | **0.03** |
| FVC z-score | 0.01 ± 0.93 | | 0.38 ± 0.97 | 0.19 ± 1.03 | 0.15 |
| **HPX-MRI DATA** | **Breathless LC**  **(n=72)** | | **Non-breathless LC (n=35)** | **Control**  **(n=30)** |  |
| RBC:M mean z-score | -0.65 ± 1.20 | | -0.57 ± 1.24 | -0.51 ± 1.01 | 0.86 |
| RBC:M CoV | 0.25 ± 0.06 | | 0.23 ± 0.06 | 0.24 ± 0.07 | 0.54 |
| RBC:Gas mean z-score | -0.86 ± 1.08 | | -0.42 ± 1.24 | -0.51 ± 1.54 | 0.16 |
| RBC:Gas CoV | 0.35 ± 0.12 | | 0.32 ± 0.06 | 0.35 ± 0.09 | 0.37 |
| **MRI PERFUSION DATA** | **Breathless**  **(n=59)** | | **Non-breathless LC (n=28)** | **Controls (n=17)** |  |
| MTT median (seconds) | 6.41 ± 1.30 | | 5.77 ± 1.39 | 6.33 ± 0.98 | 0.09 |
| MTT IQR (seconds) | 1.39 ± 0.51 | | 1.27 ± 0.50 | 1.31 ± 0.40 | 0.53 |

**Supplementary Table S1.** Questionnaire data, lung function data, HPX-MRI data and MRI perfusion data for LC participants (breathless and non-breathless) and controls. Parametric data expressed in mean ± standard deviation. P-values derived from ANOVA for parametric data, Mann-Whitney U for non-parametric data and Chi-square or fisher-exact tests for categorical data. *Abnormal TL_CO_ z-score was defined as less than ±1.64.

*Abbreviations:* CoV - Coefficient of variation; EQ-5D-5L - EuroQol-5 Dimension-5 Health today visual analogue score; FACIT-F - Functional assessment of chronic illness therapy-fatigue; FEV_1_ - Forced expiratory volume in 1 second; FVC - Forced vital capacity; GAD-7–Generalised Anxiety Disorder Questionnaire; IQR - Interquartile range; K_CO_ - Carbon monoxide transfer coefficient; LC – Long COVID; MTT - Mean transit time; RBC:M - Red blood cell to membrane ratio; RBC:Gas - Red blood cell to gas ratio; TL_CO_ - Diffusing capacity of the lungs for carbon monoxide; Va - Alveolar volume.

| Routine Laboratory bloods | Breathless LC (n=80) | Non-breathless LC (n=37) | Controls (n=34) | *P-v*alue |
| --- | --- | --- | --- | --- |
| Full blood count |  |  |  |  |
| Haemoglobin  (g/dL) | 144.1 ± 14.2 | 141.9 ± 10.3 | 138.1 ± 10.3 | 0.09 |
| Platelet count (x10^9/L) | 255.4 ± 67.9 | 259.8 ± 62.4 | 262.7 ± 54.6 | 0.85 |
| Haematocrit  (L/L) | 0.4 ± 0.0 | 0.4 ± 0.0 | 0.4 ± 0.0 | 0.30 |
| Renal function |  |  |  |  |
| Sodium  (mmoL/L) | 139.4 ± 2.1 | 139.3 ± 2.7 | 138.9 ± 1.8 | 0.61 |
| Potassium  (mmoL/L) | 4.2 ± 0.3 | 4.2 ± 0.3 | 4.1 ± 0.3 | 0.25 |
| Urea  (mmoL/L) | 5.0 ± 1.6 | 4.6 ± 1.2 | 4.7 ± 1.2 | 0.26 |
| Creatinine  (µmoL/L) | 72.1 ±15.4 | 69.2 ± 14.8 | 67.9 ± 10.5 | 0.34 |
| eGFR (mL/min/1.73m^2^) | 90.0  (82.5 – 90.0) | 90.0  (90.0 – 90.0) | 90.0  (86.0 – 90.0) | 0.09 |
| Liver function |  |  |  |  |
| Bilirubin  (µmoL/L) | 10.0 ± 5.8 | 10.0 ± 4.8 | 9.6 ± 5.0 | 0.93 |
| ALT  (IU/L) | 25.5 ± 16.9 | 24.50 ± 12.0 | 23.2 ± 11.9 | 0.76 |
| ALP  (IU/L) | 69.8 ± 21.0 | 66.9 ± 17.7 | 72.5 ± 22.3 | 0.55 |
| Albumin  (g/L) | 43.5 ± 3.6 | 44.7 ± 3.1 | 43.2 ± 2.6 | 0.13 |
| Biochemistry |  |  |  |  |
| CRP  (mg/L) | 1.9  (0.8 – 3.2) | 1.2  (0.7 – 3.5) | 2.6  (0.6 – 3.8) | 0.67 |
| NT-proBNP  (ng/L) | 59.3  (37.7 – 86.1) | 45.6  (29.8 – 78.2) | 53.7  (27.2 – 66.6) | 0.22 |
| *Missing data (n)* | *0* | *0* | *5* |  |

**Supplementary Table S2.** Baseline blood tests undertaken in local clinical National Health Service laboratories in Sheffield and Oxford. Parametric data expressed in mean ± standard deviation, non-parametric data expressed in median (interquartile range). P-values derived from ANOVA for parametric data and Kruskal-Wallis for non-parametric data. Missing data due to samples not sent to general biochemistry laboratory for testing.

*Abbreviations:* ALP - Alkaline Phosphatase; ALT - Alanine Transaminase; CRP - C-reactive protein, eGFR - Estimated glomerular filtration rate; LC – Long COVID; NT-pro BNP - N-terminal pro-B-type natriuretic peptide.

| PROTHROMBOTIC BIOMARKERS | LC  (n=117) | Control  (n=34) | *P-*value |
| --- | --- | --- | --- |
| **Coagulation/Fibrin formation** |  |  |  |
| Fibrinogen (g/L) | 2.90 (2.50 – 3.30) | 2.80 (2.60 – 3.38) | 0.83 |
| D-dimer (µg/L FEU) | 214 (190 – 353) | 224 (190 – 309) | 0.86 |
| **Endothelial activation** |  |  |  |
| von Willebrand factor (IU/mL) | 0.90 (0.68 – 1.19) | 0.93 (0.73 – 1.18) | 0.57 |
| Soluble thrombomodulin (ng/mL) | 4.15 (3.52 – 4.72) | 4.08 (3.76 – 4.36) | 0.61 |
| **Platelet activation** |  |  |  |
| sCD40L (ng/mL) | 0.10 (0.08 – 0.14) | 0.13 (0.09 – 0.15) | 0.07 |
| P-selectin (ng/mL) | 38.09 (32.66 – 46.24) | 40.69 (34.82 – 47.45) | 0.60 |
| Urinary 11-dhTXB_2_/creatinine (pg/mg) | 2755.7  (1822.3 – 3623.6) | 3101  (2249 – 4027) | 0.19 |
| **NETosis** |  |  |  |
| Myeloperoxidase (ng/mL) | 13.49 (10.78 – 17.04) | 15.27 (13.51 – 18.66) | 0.08 |
| Citrullinated H3 (ng/mL) | 0.31 (0.16 – 0.55) | 0.30 (0.16 – 0.51) | 0.95 |
| **Fibrinolysis** |  |  |  |
| PAI-1 (ng/mL) | 8.38 (6.07 – 11.65) | 8.34 (5.25 – 11.85) | 0.92 |
| 50% clot lysis time (minutes) | 42.68 (38.00 – 50.26) | 40.53 (35.58 – 48.35) | 0.24 |

**Supplementary Table S3.** Prothrombotic biomarkers in LC participants (grouped) and controls. Non-parametric data expressed in median (interquartile range). P-values derived from Kruskal-Wallis. Abbreviations: 11-dhTxB_2_ - 11-dehydro-thromboxane B_2;_ LC – Long COVID; NET - neutrophil extracellular trap; PAI-1 - Plasminogen activator inhibitor-1.

| Demographics | Breathless LC  (n=27) | Non-breathless LC  (n=14) | Controls  (n=14) | P-value |
| --- | --- | --- | --- | --- |
| Mean age (years) | 46 ± 11 | 47 ± 10 | 45 ± 12 | 0.84 |
| Female | 18 (66.7%) | 11 (78.6%) | 9 (64.3%) | 0.75 |
| BMI (kg/m^2^) | 28.7 ± 5.4 | 29.9 ± 6.0 | 29.3 ± 7.1 | 0.83 |
| Days post-COVID infection | 570 (396 – 825) | 501 (457 – 837) | 542 (498 – 589) | 0.98 |
| FEV_1_ z-score | -0.03 ±0.92 | 0.40 ± 1.08 | 0.23 ± 0.76 | 0.36 |
| FVC z-score | 0.02 ± 0.94 | 0.36 ± 1.02 | 0.38 ± 1.03 | 0.42 |
| TL_CO_ z-score | -0.60 ± 0.67 | -0.05 ± 1.04 | -0.07 ± 1.20 | 0.11 |

**Supplementary Table S4.** Demographics and lung function data for LC participants (breathless and non-breathless) and controls in the fibrin network and fibrin(ogen) microaggregate sub-analysis. Parametric data expressed in mean ± standard deviation; non-parametric data expressed in median (interquartile range). P-values derived from ANOVA for parametric data and Kruskal-Wallis for non-parametric data.

*Abbreviations:* BMI - Body mass index, FEV_1_ - Forced expiratory volume in 1 second; FVC - Forced vital capacity; LC – Long COVID; TL_CO_ - Diffusing capacity of the lungs for carbon monoxide

| Fibrin(ogen) microaggregate characteristics | Breathless LC (n=27) | Non-breathless LC (n=14) | Controls  (n=14) | P -value |
| --- | --- | --- | --- | --- |
| Number of microaggregates >100μm^2^ | 23.9 ±14.1 | 26.3 ±11.6 | 19.8 ±8.5 | 0.31 |
| Area of microaggregates (μm^2^) | 154.8 ± 95.1 | 177.5 ± 101.1 | 193.3 ± 148.6 | 0.10 |
| Fibrin fiber density (fibers/100μm) | 38.0 ± 11.0 | 43.6 ± 12.3 | 47.9 ± 15.0 | 0.22 |

**Supplementary Table S5.** Fibrin(ogen)microaggregate characteristics: number of microaggregates greater than 100µm^2^; area of the microaggregates and fibrin fiber density between participants with LC (breathless and non-breathless) and controls. Parametric data presented as mean ± standard deviation. P-values derived from Kruskal Wallis Test. *Abbreviations*: LC – Long COVID.

| Fibrin(ogen) microaggregate characteristics | Long Covid (n=41) | Controls  (n=14) | P -value |
| --- | --- | --- | --- |
| Number of microaggregates >100μm^2^ | 25.00 ± 13.7 | 19.8 ±8.5 | 0.30 |
| Area of microaggregates (μm^2^) | 143.20 ± 89 | 193.3 ± 148.6 | 0.46 |
| Fibrin fiber density (fibers/100μm) | 41.90 ± 12.0 | 47.9 ± 15.0 | 0.17 |

**Supplementary Table S6.** Fibrin(ogen)microaggregate characteristics: number of microaggregates greater than 100µm^2^; area of the microaggregates and fibrin fiber density between participants with Long Covid and controls. Parametric data presented as mean ± standard deviation. P-values derived from Kruskal Wallis Test.
