# Supplementary material for: No evidence of hypercoagulability, altered fibrinolysis, or increased fibrin(ogen) microaggregates in never‐hospitalized long COVID
Source: Hemasphere. 2026 Oct 19;10(10):e70501. doi: 10.1002/hem3.70501 (PMC13680132; doi:10.1002/hem3.70501)
Supplement: Supplementary file 3 [file HEM3-10-e70501-s003.docx]

**Supplementary Digital Content**

# **Supplementary methods**

*Study design and participants*

This multicentre observational cohort study was approved by the South Central Research Ethics Committee (21/SC/0398); International Standard Randomised Controlled Trial Number (ISRCTN14304264). It recruited patients from four UK specialist LC centres. The study protocol and inclusion/exclusion criteria has previously been published[1].

In brief, eligible participants were ≥18 years and had never been hospitalised with acute COVID-19. For participants with LC, the diagnosis was based on the NICE definition[2], and made following comprehensive evaluation in a dedicated multidisciplinary LC clinic with exclusion of other possible causes for symptoms. LC participants were divided into ‘breathless’ and ‘non-breathless’ groups based on clinical evaluation by the recruiting physician.

Controls were identified through poster advertisement and were age- and sex-matched. Controls had had prior COVID-19 (PCR or lateral flow test proven) but were fully recovered.

Following screening, participants with significant abnormalities identified on chest computed tomography (CT), those with evidence of airflow obstruction (forced expiratory volume in 1 second/forced vital capacity (FEV_1_/FVC) ratio of <0.7), current smokers, or those on anticoagulation or anti-platelets were excluded.

*EXPLAIN study: clinical data collection and respiratory investigations*

Investigations were undertaken on study visits. These included dissolved phase HPX-MRI, DCE-MRI, chest CT, pulmonary function tests (PFTs) comprising: Forced Expiratory Volume in 1-second (FEV_1_, indicator of airway calibre, reduced in airways disease); Forced Vital Capacity (FVC, indicator of lung capacity); Transfer factor of the Lung for Carbon monoxide (TL_CO_, measure of gas diffusion across the alveolar membrane) adjusted for lung capacity measured by the Alveolar Volume (VA); and carbon monoxide transfer coefficient (K_CO_, measure of gas transfer per unit volume). Questionnaires comprised: FACIT-Fatigue-questionnaire focused around fatigue, with lower scores indicating more severe fatigue; Dyspnoea-12 (D12), questionnaire assessing physical and affective components of breathlessness, with higher scores indicating more severe breathlessness; Generalised Anxiety Disorder-7 (GAD7) and general health today questionnaire (EuroQoL-VAS), where higher scores indicate better health.

Dissolved phase HPX-MRI was acquired as previously described [3]. Pulmonary gas transfer between the air space and red blood cells (RBC:Gas) and between the alveolar-capillary membrane and the red blood cells (RBC:Membrane) were calculated and presented as z-scores based on normative data [4]. DCE-MRI was acquired as previously described [5] and maps of pulmonary mean transit time (MTT) were calculated for each subject.

Routine laboratory tests (renal and liver function, full blood count, C-reactive protein (CRP) and NT-proBNP (B-type natriuretic peptide) were undertaken as part of the main study and analysed at the local clinical National Health Service laboratories.

All the haemostasis sub-study laboratory testing was undertaken in Oxford. For the haemostasis sub-study, different aspects of haemostasis and thrombosis were assessed using standard laboratory assays: coagulation/fibrin formation (Clauss fibrinogen, D-dimer), endothelial activation (von Willebrand factor, soluble thrombomodulin), platelet activation (sCD40L, P-selectin, urinary 11-dhTXB2/creatinine ratio), NETosis (myeloperoxidase, citrullinated H3) and fibrinolysis (plasminogen activator inhibitor-1, 50% clot lysis time) and fibrin fiber density following *in vitro* clot formation; as well as quantifying the presence of relatively novel fibrin(ogen)-cell membrane aggregates using a previously reported technique[6]. For these samples, blood was collected in 2.7ml blue citrate (3.2%) BD Vacutainer™ tubes using standard venepuncture technique by qualified practitioners. Platelet poor plasma was obtained by centrifugation of whole blood samples at room temperature for 15 minutes at 3000xg, aliquoted and frozen at -80°C within 4 hours of collection. Urine sample was collected and aliquoted into two 1ml samples which were frozen. Following processing, frozen samples were shipped to Oxford on dry ice for participants recruited at other sites.

*Determination of plasma and urine protein concentrations*

Concentrations of D-dimer and Clauss fibrinogen were determined using Sysmex CS5100 analyser (Siemens Clauss fibrinogen reagent, Siemens Innovance D-dimer reagent). Other plasma proteins were quantified using commercial ELISAs according to manufacturers' guidelines: human von Willebrand factor (Abcam, ab223864); human soluble thrombomodulin (Abcam, ab214029); human plasminogen activator inhibitor (PAI)-1 ELISA (Abcam, ab269373); human P-selectin (Proteintech, KE00064); human soluble (s) CD40L (Abcam, ab196268); citrullinated Histone H3 (Cayman Chemical, 501620); human myeloperoxidase (Abcam, ab272101). Urinary thromboxane B2 was quantified using the human 11-dehydro-Thromboxane B2 ELISA (Cayman Chemical, 519510) as per manufacturer instructions. Urine creatinine was measured using an enzymatic method utilising creatininase on an Abbott Architect c16000 clinical chemistry analyser (Abbott Laboratories, Maidenhead, UK).

*Clot lysis*

Study participant’s platelet-poor plasma 30% (v/v), 16 µM phospholipids (Rossix, Sweden), and 90 pM tissue plasminogen activator (Sigma-Aldrich, USA) in Tris-buffered saline with Tween 20 (10 mM TRIS, 140mM NaCL, 0.01% Tween 20, pH 7.40) were added to 96-well flat-bottom plates. CRYOcheck pooled normal plasma (PNP; PrecisionBiologic) was used as a control, and samples were analysed in triplicate. Clotting was initiated with 10.6mM/mL CaCl_2_. Clot formation and lysis were monitored by light absorbance (405 nm), measured every 60 s for 480 minutes using the Multiskan Ascent microplate reader (ThermoScientific), and the optical readings collected via the Ascent Software (v2.6).  The absorbance-time curves were generated and their associated 50% clot lysis times were calculated using the Longstaff C, 2019, Shiny App for analysing clotting and lysis curves, version 1.2.3 [7].

*Confocal microscopy of fibrin structure and fibrin(ogen) microaggregates*

Plasma samples were thawed at 37°C in a water-bath for 10 minutes (to prevent cold-agglutinin activation). Plasma was diluted 1:4 in tris-buffered saline, mixed with AlexaFluor488-labelled human fibrinogen and DiOC18(3) (final concentrations 50ug/ml, 0.0012ug/ml respectively, both from Life Technologies Ltd.) and left to incubate at room temperature for 10 minutes.

To assess fibrin(ogen) microaggregates, the plasma mix was loaded into the channel of an uncoated µ-slide (VI 0.4 slides, Ibidi GmbH, Germany) and then imaged. Microaggregate imaging was performed without addition of thrombin or calcium, allowing assessment of fibrin(ogen)–cell membrane microaggregates present in plasma prior to any in‑vitro induction of coagulation.

To assess fibrin clot structure, first clotting was triggered in the plasma mix with human alpha thrombin (0.1units/ml, Merck, UK) and 10mM CaCl2, and then the sample was loaded into the channel of an uncoated µ-slide (VI 0.4 slides, Ibidi GmbH, Germany). These slides were kept overnight at room temperature in dark humidity chambers before imaging.

All channels were imaged using a Zeiss LSM880 confocal microscope with 40× oil immersion objectives (Carl Zeiss, Welwyn Carden City, UK). For fibrin(ogen) microaggregate analysis, each channel was systematically imaged using a tile scan (total area 1653µm^2^) in three locations (top/middle/bottom of each channel). ImageJ software (Wayne Rasband, NIH) was used to analyse images. Images were made binary and the “Analyze Particles” macro was used to determine number and area of aggregates. Any microaggregates of 5µm^2^ or larger were identified on each tile, and quantified in number and size area. For fibrin network analysis, Z-stack (20 µm, 30 slices) images were taken in 3 areas per clot of large microaggregates and surrounding network. Images were projected to a single plane. Image J was used to determine the density of the fibrin clot structure. An in-house macro was used to calculate the number of fibers per 100 μm. The images were taken and analysed by a blinded operator.

*Statistical analysis*

Statistical analysis was performed using statistical software R version 4.2.2 and R studio (The R Project; The R Foundation) and GraphPad Prism Software (Version 10.1.2). Normally distributed data are expressed as mean ± standard deviation and analysed using unpaired t-test or one-way ANOVA. Otherwise, data are expressed as median ± inter-quartile range (IQR) (Mann-Whitney U tests or Kruskal-Wallis). The clot-lysis curve and 50% clot lysis time was calculated using Shiny App (version 0.6). Confocal microscopy images were analysed using ImageJ 1.51w and an in-house macro. Correlations were calculated for serum biomarkers and lung function metrics: Transfer factor for lung carbon monoxide (TL_CO_), Red blood cell:Membrane (RBC:M) and RBC:Gas z-scores and pulmonary perfusion MTT. Z-scores outside -1.64 and +1.64, which correspond to the 5^th^ and 95^th^ percentile limits were taken as abnormal[8]. Questionnaire scores for breathing (Dyspnoea-12), fatigue (FACIT-F) and general health (EuroQoL-VAS) were also included. Correlations were summarised using Spearman’s correlation coefficient, with 95% confidence intervals derived using non-parametric bootstrap. A value of p <0.05 was considered statistically significant for all tests.

Supplementary References

1.	Ng, K.L., et al. *Using Hyperpolarised Xenon MRI to Explore Breathlessness in Long COVID.* Accepted for publication by the European Respiratory Journal 2026. Doi:10.1183/23120541.00040-2026.

2.	*National Institute of Clinical Excellance (NICE). COVID-19 rapid guideline: managing the long-term effects of COVID-19. NICE guideline NG188. Published: 18 December 2020. Last updated: 25 January 2024 [Accessed 25 March 2026].*

3.	Collier, G.J., et al., *Dissolved (129) Xe lung MRI with four-echo 3D radial spectroscopic imaging: Quantification of regional gas transfer in idiopathic pulmonary fibrosis.* Magn Reson Med, 2021. **85**(5): p. 2622-2633.

4.	Collier, G.J., et al., *Age, sex, and lung volume dependence of dissolved xenon-129 MRI gas exchange metrics.* Magn Reson Med, 2024. **92**(4): p. 1471-1483.

5.	Saunders, L.C., et al., *Longitudinal Lung Function Assessment of Patients Hospitalized With COVID-19 Using (1)H and (129)Xe Lung MRI.* Chest, 2023. **164**(3): p. 700-716.

6.	Baker, S.R., et al., *Plasma from patients with pulmonary embolism show aggregates that reduce after anticoagulation.* Commun Med (Lond), 2023. **3**(1): p. 12.

7.	Longstaff C. *Analysis of clotting and lysis data. https:// drclongstaff.github.io/shiny-clots/#lysis.* [Accessed 27 March 2025].

8.	Stanojevic, S., et al., *ERS/ATS technical standard on interpretive strategies for routine lung function tests.* Eur Respir J, 2022. **60**(1).
