# Supplementary material for: No evidence of hypercoagulability, altered fibrinolysis, or increased fibrin(ogen) microaggregates in never‐hospitalized long COVID
Source: Hemasphere. 2026 Oct 19;10(10):e70501. doi: 10.1002/hem3.70501 (PMC13680132; doi:10.1002/hem3.70501)
Supplement: Supplementary file 4 [file HEM3-10-e70501-s004.docx]

**Figure S2.** Plots to examine potential associations between prothrombotic biomarkers with symptoms measured via patient‐reported outcome measures (dyspnoea‐12, FACIT‐F, and EuroQoL‐VAS), gas transfer (TL_CO_ *z*‐score), and HPX‐MRI metrics of diffusion (RBC:M mean *z*‐score, RBC:Gas mean *z*‐score), and perfusion MRI measures of pulmonary mean transit time (MTT in seconds). Abbreviations: 11‐dhTxB_2_, 11‐dehydro‐thromboxane B_2_; FACIT‐F, functional assessment of chronic illness therapy‐fatigue; MPO, myeloperoxidase; PAI‐1, plasminogen activator inhibitor‐1; RBC:Gas, red blood cell to gas ratio; RBC:M, red blood cell to membrane ratio; sCD40L, soluble CD40 ligand; TL_CO_, diffusing capacity of the lungs for carbon monoxide; VAS, visual analog scale.
